# Inhibition of mitochondrial permeability transition pore restores the cardioprotection by postconditioning in diabetic hearts

**DOI:** 10.1186/s40200-014-0106-1

**Published:** 2014-11-18

**Authors:** Moslem Najafi, Safar Farajnia, Mustafa Mohammadi, Reza Badalzadeh, Naser Ahmadi Asl, Behzad Baradaran, Mohammad Amani

**Affiliations:** Drug Applied Research Center, Tabriz University of Medical Sciences, Tabriz, Iran; Biotechnology Research Center, Tabriz University of Medical Sciences, Tabriz, Iran; Department of Physiology, Faculty of Medicine, Tabriz University of Medical Sciences, Tabriz, Iran; Immunology Research Center, Tabriz University of Medical Sciences, Tabriz, Iran

**Keywords:** Reperfusion injury, Diabetes, Postconditioning, mPTP, Cardioprotection

## Abstract

**Background:**

Cardiovascular risk factors, including diabetes mellitus may attenuate the cardioprotection by postconditioning. This study aimed to investigate the cardioprotective effect of ischemic-postconditioning (IPostC) against ischemia/reperfusion injury in normal and chronically type-1 diabetic rats and the effect of mitochondrial permeability transition pore (mPTP) inhibition in this field.

**Methods:**

Diabetes was induced by a single intra-peritoneal injection of streptozotocin (50 mg/kg) in Wistar male rats (250-300 g). After 8 weeks, the hearts of control and diabetic animals were isolated and mounted on a constant-pressure Langendorff apparatus. All hearts were subjected to 30 min regional ischemia followed by 45 min reperfusion (by occluding and re-opening of LAD coronary artery, respectively). At the end of ischemia, the hearts received IPostC, cyclosporine-A, or both or none of them. Myocardial creatine-kinase (CK) release as an index of tissue injury was measured spectrophotometery in coronary effluent in reperfusion phase. Infarct size was identified by triphenyltetrazolium chloride staining. Heart rate, left ventricular end-diastolic pressure (LVEDP), LV systolic pressure (LVSP), rate-pressure product (RPP) and coronary flow were recorded throughout the experiment.

**Results:**

IPostC, applied at the onset of reperfusion, failed to improve myocardial LVEDP and RPP, or reduce tissue damage indicated by infarct size and CK release in diabetic hearts, while it significantly recovered these parameters toward the pre-ischemic values in control hearts (*P* < 0.05). In contrast, with simultaneous inhibition of mPTP using cyclosporine-A, the cardioprotective effects of IPostC on myocardial hemodynamics, infarct size and CK release were significantly restored in diabetic hearts (*P* < 0.05).

**Conclusions:**

The loss of cardioprotection by IPostC in diabetic state can be overcome by increasing the potency of protective IPostC through its co-application with mPTP inhibition.

## Introduction

Myocardial reperfusion injury is a condition in which restoring the blood supply to the tissue after a long period of ischemia or lack of oxygen further damage the myocardium and leads to myocardial infarction (MI) [1]. Several cardioprotective interventions including preconditioning and postconditioning have been examined in experimental animals and humans, with promising results [2-4]. Ischemic postconditioning (IPostC) is a strategy by which the repetitive very short episodes of ischemia and reperfusion (I/R) applied at the onset of reperfusion confers significant cardioprotective effects and limit MI [4]. Unfortunately, the majority of the studies investigating the protective effects of IPostC have been performed in models of healthy animals without underlying risk factors and comorbidities, including diabetes mellitus. This is in contrast to human populations with ischemic heart disease (IHD) who also have other comorbidities that affect the tolerance to I/R injury and the protective strategies [5-7].

Diabetes mellitus is a metabolic disorder characterized by hyperglycemia and insufficient secretion or action of endogenous insulin in the body [8] and is associated with a higher risk of IHD including MI. Diabetic mellitus might affect the myocardium in diabetic patients causing changes at the cellular level, which, in turn, lead to a wide range of structural and biochemical abnormalities eventually leading to systolic and diastolic dysfunction [9].

Currently, there is little knowledge about the interaction of chronic diabetes with cardioprotective effect of IPostC and the mechanisms of this interaction are also not understood completely. One of the critical determinants of the reperfusion injury is the mitochondrial permeability transition pore (mPTP) which opens during reperfusion injury due to oxidative stress, Ca^2+^overload, decreased ATP levels, and increased matrix pH [10]. The mPTP has also been linked to the pathophysiology of diabetes-induced alterations in cellular mechanisms [11]. To our knowledge, mPTP play a crucial role in postconditioning. IPostC has been suggested to close this non-selective ion channel via involving a variety of intracellular mediators and thereby confer a cardioprotection [12]. Cyclosporine-A (CsA) is an immunosuppressive agent which also inhibits the mPTP opening at reperfusion and previous studies have revealed that it is possible to protect the heart against I/R injury by CsA administration at the onset of reperfusion [10,12], however, it has not been feasible in diabetic hearts as reported by some studies [13] and restoring the protection in diabetes is of clinically important.

Therefore, because of the clinical importance of the interaction of chronic diabetes with cardiovascular diseases and cardioprotective mechanisms of IPostC and due to the lack of enough information in this subject, this study investigated the cardioprotective effect of IPostC against I/R injury in normal and chronically streptozotocin-induced diabetic rats and the effect of simultaneous inhibition of mPTP in this field.

## Materials and methods

### Animals

Male Wistar rats (250–300 g body weight) were used in this study and had free access to food and water at all times before the start of the experiments. They were housed in laboratory room with a 12-h dark and 12-h light cycle. This study was performed in accordance with the Guide for the Care and Use of Laboratory Animals published by the US National Institutes of Health (NIH publication No 85–23, revised 1996) and was approved by the local Animal Care and Ethics Committee.

### Induction of diabetes

Diabetes was induced by a single intra-peritoneal injection of streptozotocin (50 mg/kg body weight; Sigma-Aldrich, Germany) in diabetic groups. Development of the diabetes was confirmed 72 h later by measuring blood glucose levels using a glucometer device through the sampling of blood with small scratching of tail of rats. The animals with blood glucose levels higher than 300 mg/dl were considered as diabetic [14,15]. After 8 weeks of the disease, the diabetic animals as well as the age-matched controls were sacrificed and all experiments were performed in isolated perfused hearts.

### Surgical preparation, isolated heart perfusion and hemodynamics measurements

Surgical preparation was performed as described previously [16,17]. All animals were heparinized (500 IU) and anesthetized with a mixture of ketamine (60 mg/kg) and xylosine (10 mg/kg) intra-peritoneally. The hearts were rapidly excised, then mounted on the Langendorff perfusion apparatus and retrogradely perfused via the aorta with a Krebs–Henseleit (K-H) solution (in mM/l: NaCl 118; KCl 4.7; CaCl_2_ 2.5; MgSO_4_. 1.2; NaHCO_3_ 25; KH_2_PO_4_ 1.2; Glucose 11.1; all materials from Merck, Germany) at a constant perfusion pressure of 75 mmHg and pH 7.4. The perfusion solution was gassed with a mixture of 95% O_2_, 5% CO_2_ at 37°C. A water-filled latex balloon was inserted into the left ventricle through an incision in the left atrium. The balloon volume was adjusted to produce 5–10 mmHg of end-diastolic pressure at the onset of experimen in all experimental groups. Hemodynamic data from the balloon were digitized and continuously recorded by a data acquisition system (PowerLab; ADInstruments, Australia), displayed on a monitor and analyzed using Chart 7.3 for windows Software. The heart rate (HR), left ventricular end diastolic pressure (LVEDP), left ventricular systolic pressure (LVSP), and rate-pressure product (RPP = LVSP × HR) were recorded. The coronary flow (CF) was measured by timed-collection of coronary effluent in certain time points.

### Induction of regional ischemia and reperfusion

At the onset of experiment, a 5–0 silk thread was placed around the left anterior descending (LAD) coronary artery, close to its origin. After stabilization period (15 min), all hearts were subjected to regional ischemia for 30 min followed by reperfusion for 45 min. Regional ischemia and reperfusion were induced by occluding and re-opening of LAD, respectively. An immediate fall in coronary flow at the onset of index ischemia (to about 30-40% of its baseline value) and its recovery upon reperfusion served as evidences of effective coronary occlusion and reperfusion [18]. Ischemic postconditioning (IPostC) strategy in this study was three cycles of 30s reperfusion and ischemia (3 cycles of 30s R/I), respectively, immediately at the onset of reperfusion.

### Exclusion criteria

During the 8-week of diabetes induction and before the starting the experiment on the Langendorff setting, the diabetic animals with blood glucose levels lower than 300 mg/dl were excluded from the study. Moreover, in the Langendorff setting, the isolated hearts were excluded from the experiment if their baseline LVDP was lower than 70 mmHg in control rats or if they showed severe arrhythmias or if there were no effective coronary occlusion and reperfusion.

### Experimental protocols

The animals were divided into eight groups (n = 6 each) as following:

1. Control (C); in which after surgical preparation and stabilization period, the isolated hearts of non-diabetic animals were subjected to 30 min ischemia and 45 min reperfusion.
2. Control with ischemic postconditioning (C + IPostC); in which the condition was similar to the control group except that at the onset of reperfusion, the hearts were received 3 cycles of 30s R/I.
3. Control with cyclosporine-A (C + CsA); in which the condition was similar to the control group except that 5 min before the onset of reperfusion up to 10 min of reperfusion, the hearts were perfused with a K-H solution containing 0.01 mM CsA.
4. Control with ischemic postconditioning plus cyclosporine-A (C + IPostC + CsA); in which the condition was similar to the control group except that 5 min before the onset of reperfusion up to 10 min of reperfusion, the hearts were perfused with a K-H solution containing CsA, and at the onset of reperfusion, they were received 3 cycles of 30s R/I.
5. Diabetic (D); in which after surgical preparation and stabilization period, the isolated hearts of 8-week diabetic animals were subjected to 30 min ischemia and 45 min reperfusion.
6. Diabetic with ischemic postconditioning (D + IPostC); in which the condition was similar to the diabetic group except that at the onset of reperfusion, the hearts were received 3 cycles of 30s R/I.
7. Diabetic with cyclosporine-A (D + CsA); in which the condition was similar to the diabetic group except that 5 min before the onset of reperfusion up to 10 min of reperfusion, the hearts were perfused with a K-H solution containing CsA.
8. Diabetics with ischemic postconditioning plus cyclosporine A (D + IPostC + CsA); in which the condition was similar to the diabetic group except that 5 min before the onset of reperfusion up to 10 min of reperfusion, the hearts were perfused with a K-H solution containing CsA, and at the onset of reperfusion, they were received 3 cycles of 30s R/I.

### Measurement of creatine kinase

The coronary effluent was collected for 10 min after initiation of reperfusion for measuring the activity of creatine kinase (CK) as an index for tissue injury. The CK activity in the coronary effluent was measured by an automatic biochemistry analyzer using a commercially available kit according to the manufacturer’s instructions, and the values was normalized to whole volumes of the effluent in each heart. The absorbance of the solution was detected at 340 nm by spectrophotometery. The results were reported in U/L.

### Measurement of infarct size

In another series of experiment with a similar grouping with a reperfusion period of 60 minutes, infarct sizes were identified as described previously. At the end of the experiment, the LAD coronary artery was then re-occluded at the same site and 3 to 4 mL of 0.25% Evans blue dye was infused via the aortic root into the coronary system. These identify the area at risk as unstained and non-ischemic part of the myocardium as blue. The heart was then frozen, cut into thin slices (2 mm) from the apex to the base and incubated (for 10 to 15 minutes at 37°C) in buffered 1% 2,3,5-triphenylte-trazolium chloride (TTC) to identify viable myocardium as red stained while necrotic (infarct) tissue remains pale gray. The Left ventricular, risk zones and infarcted areas were determined using a computerized planimetry (Summa Sketch III, SummaGraphics, USA by one observer blinded to the study group. The infarct sizes were expressed as a percentage of the left ventricular risk zones and the volumes were calculated by multiplying the areas by slice thickness and summing them for each heart.

### Statistical analysis

All values were expressed as means ± SEM. The differences between groups were analyzed using repeated measures ANOVA with bonferroni test or One-way ANOVA followed by Tukey's post hoc test. Differences were considered statistically significant when *P* < 0.05.

## Results

### Baseline parameters

The baseline hemodynamics recorded before the induction of ischemia in experimental subgroups are shown in Table 1. At the end of 8 weeks of diabetes as a model of chronic disease in the present study, the diabetic animals showed a marked myocardial dysfunction and cardiomyopathy, as revealed by significantly reduced HR, CF, LVSP and RPP in diabetic animals as compared to those of control animals (*P* < 0.05). Although there was no significant difference of the hemodynamic parameters within control subgroups or within diabetic subgroups, these baseline parameters were significantly lower in all diabetics than controls (Table 1).

**Table 1:** **Baseline values of hemodynamic parameters in non-diabetic control and diabetic sub-groups**

**Group**	**HR (beat/min)**	**CF (ml/min)**	**LVEDP (mmHg)**	**LVSP (mmHg)**	**RPP (1/1000)**
**C**	259 ± 12	10.6 ± 0.9	7.4 ± 0.7	94 ± 6	25.2 ± 0.80
**C + IPostC**	272 ± 11	11.0 ± 0.6	7.1 ± 0.7	97 ± 5	26.5 ± 0.73
**C + CsA**	277 ± 14	12.8 ± 1.4	6.7 ± 0.6	99 ± 6	27.7 ± 0.92
**C + IPostC + CsA**	274 ± 26	11.6 ± 0.5	7.3 ± 1.1	102 ± 7	28.0 ± 1.47
**D**	198 ± 13*	7.7 ± 0.7*	8.5 ± 0.8	78 ± 8*	15.7 ± 0.93*
**D + IPostC**	192 ± 16*	9.0 ± 0.8*	7.4 ± 0.8	75 ± 8*	14.6 ± 1.41*
**D + CsA**	202 ± 12*	9.8 ± 1.2*	8.0 ± 0.6	78 ± 6*	15.0 ± 0.65*
**D + IPostC + CsA**	224 ± 18*	9.5 ± 0.9*	7.8 ± 0.9	73 ± 7*	16.6 ± 1.38*

*P < 0.05 as compared with control groups. *Abbreviation*: *"C"* control, *"C + IPostC"* control with ischemic postconditioning, *"C + CsA"* control with cyclosporine-A, *"C + IPostC + CsA"* control with ischemic postconditioning plus cyclosporine-A, *"D"* diabetic, *"D + IPostC"* diabetic with ischemic postconditioning, *"D + CsA"* diabetic with cyclosporine-A, *"D + IPostC + CsA"* diabetics with ischemic postconditioning plus cyclosporine-A. *HR* heart rate, *CF* coronary flow, *LVEDP* left ventricular end diastolic pressure, *LVSP* LV systolic pressure, and *RPP* rate-pressure product.

### CK activity

**Figure 1:**
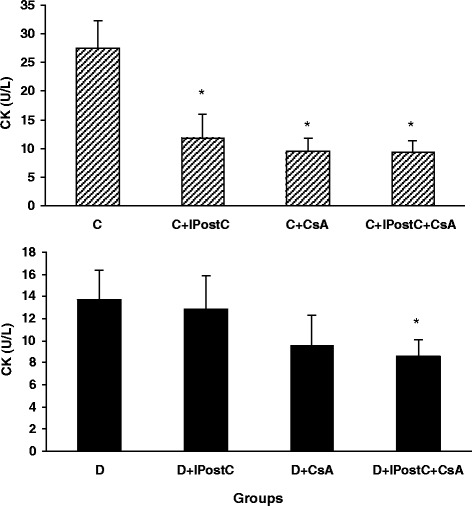
**CK activity in coronary effluent of non-diabetic control (top) and diabetic (bottom) hearts.** *P < 0.01 *vs*. group C (top) or *vs*. group D (bottom).

The levels of CK release into the coronary effluent of both control and diabetic animals were shown in Figure 1. In control hearts, ischemic postconditioning (IPostC) and cyclosporine-A (CsA), alone or in combination with each other (IPostC + CsA) significantly reduced CK levels as compared to non-treated control hearts (C) (*P* < 0.01). However, in diabetic hearts, only the combination therapy (IPostC + CsA) could significantly reduced the CK levels as compared to non-treated diabetic hearts (D) (*P* < 0.01); the CK reducing effects of IPostC alone or CsA alone were not significant in comparison with diabetic group (Figure 1).

### Infarct size

**Figure 2:**
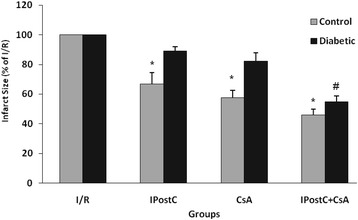
**Infarct sizes (in percentage of I/R group) in non-diabetic control (gray bars) and diabetic (dark bars) hearts.** *P < 0.05 *vs*. corresponding I/R control group; and ^#^ *P* < 0.01 *vs*. corresponding I/R diabetic group.

According to the results of Evans Blue staining, 30 min occluding of LAD produced similar risk zones or extent of ischemic tissue (which indicates the degree of ischemic stimulus) in both control and diabetic hearts and there were no significant differences in the risk zones between all groups of control and diabetic animals (averaged 43.78 ± 3.9% of LV volumes in controls vs. averaged 47.31 ± 3.7% of LV volumes in diabetics). On the other hand, thirty minutes ischemia and sixty minutes reperfusion led to the development of 41.2 ± 2.9% infarct size in control hearts vs. 39.3 ± 3.1% in diabetic hearts. Both alone treatment of IPostC or CsA significantly reduced the infarct size of non-diabetic control hearts by 33% and by 42%, respectively (*P < 0.05*). However, neither IPostC nor CsA significantly reduced the infarct size in the diabetic rats. Furthermore, in non-diabetic hearts, combined administration of IPostC with CsA had a greater and significant infarct reducing effect (by 54%) as compared with those of control untreated-hearts (*P < 0.01*). Although IPostC alone could not alter the infarct size in diabetic rats, combination of CsA and IPostC provided a significant infarct reducing effect (by 46%) in diabetic groups (*P < 0.01*) (Figure 2).

### LVEDP alterations during I/R

**Figure 3:**
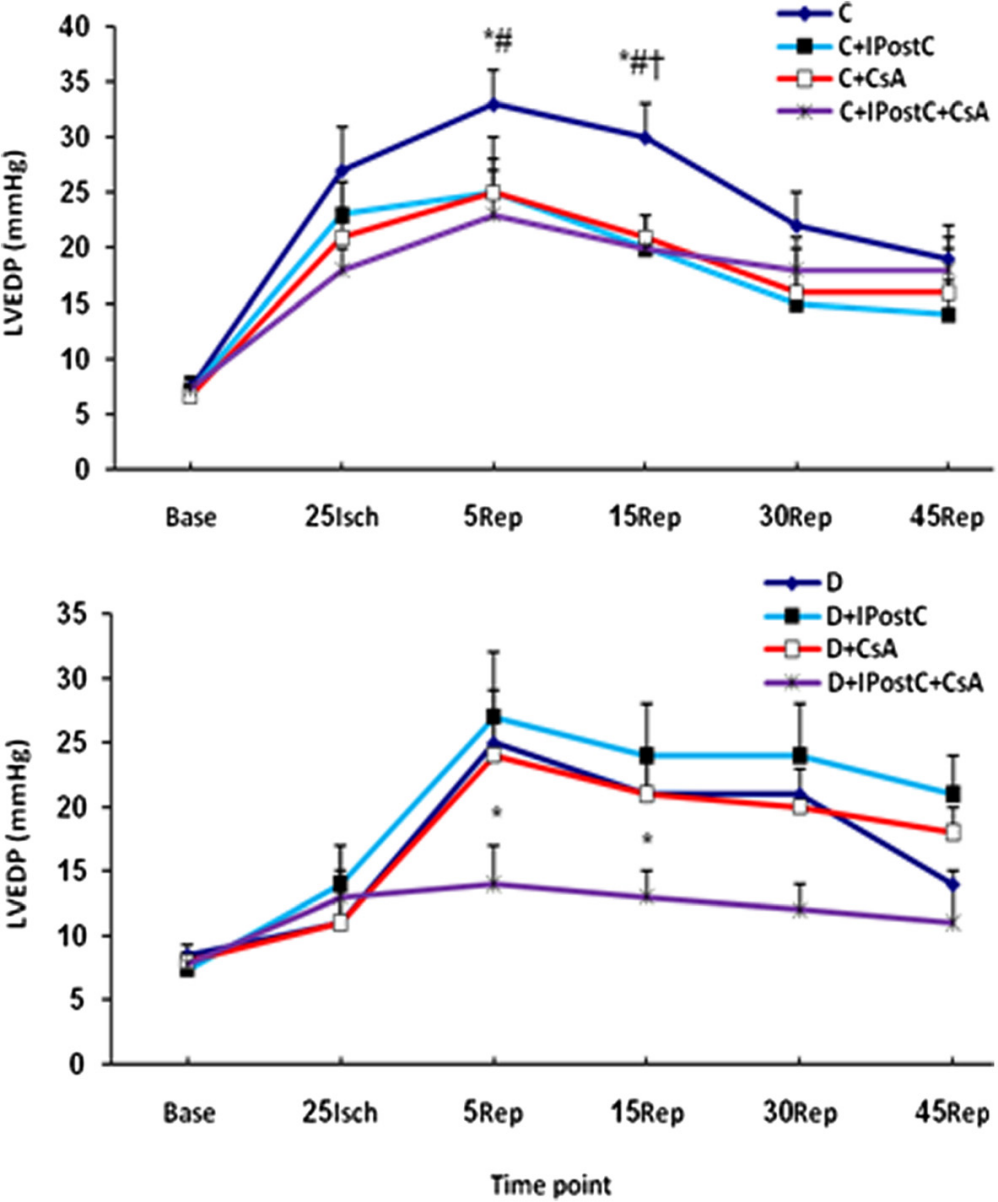
**The alterations of the left ventricular end diastolic pressure (LVEDP; in mmHg) in control (C; top) and diabetic (D; bottom) subgroups throughout the ischemia (Isch) and reperfusion (Rep) time.** The numbers in horizontal axis (time point) represent the minutes at which the values have recorded. #*P* < 0.05 difference between C + IPostC and C; †*P* < 0.05 difference between C + CsA and C; and *P < 0.05 difference between C + IPostC + CsA and C (top); or between D + IPostC + CsA and D (bottom).

Administration of IPostC or CsA, alone or in combination, significantly prevented the reperfusion-induced increase in LVEDP in control non-diabetic animals (*P* < 0.05). None of IPostC or CsA alone could reduce the LVEDP during the reperfusion phase in diabetic animals. However, concomitant administration of both IPostC and CsA completely prevented the increase in LVEDP in diabetic hearts (*P* < 0.05) (Figure 3).

### RPP alterations during I/R

**Figure 4:**
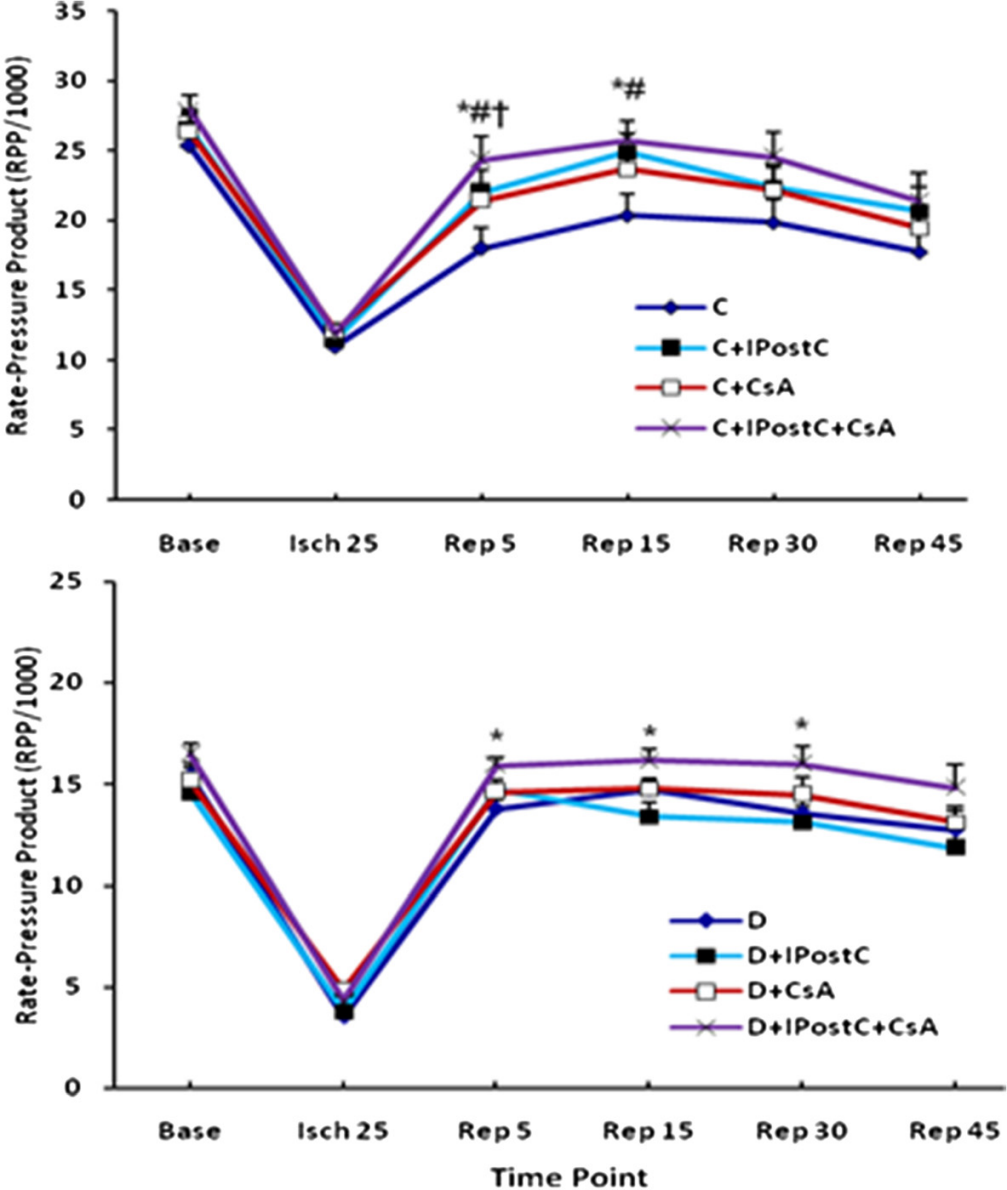
**The alterations of the myocardial rate-pressure product (RPP;% of baseline) in control (C; top) and diabetic (D; bottom) subgroups throughout the ischemia (Isch) and reperfusion (Rep) time.** #*P* < 0.05 difference between C + IPostC and C; †*P* < 0.05 difference between C + CsA and C; and *P < 0.05 difference between C + IPostC + CsA and C (top); or between D + IPostC + CsA and D (bottom).

IPostC and CsA in non-diabetic control animals significantly improved the RPP, as an index for cardiac work, at some time points during reperfusion (*P* < 0.05) (Figure 4). In contrast, both of them failed to restore the myocardial RPP in diabetic rats. Nevertheless, administration of CsA in postconditioned diabetic hearts could significantly return the RPP during reperfusion toward the pre-ischemic baseline values, similar to those in non-diabetic control hearts (*P* < 0.05). It should also be noted that there were no significant differences in RPP between three subgroups of IPostC, CsA and IPostC + CsA in control animals (Figure 4, top).

### HR alterations during I/R

**Figure 5:**
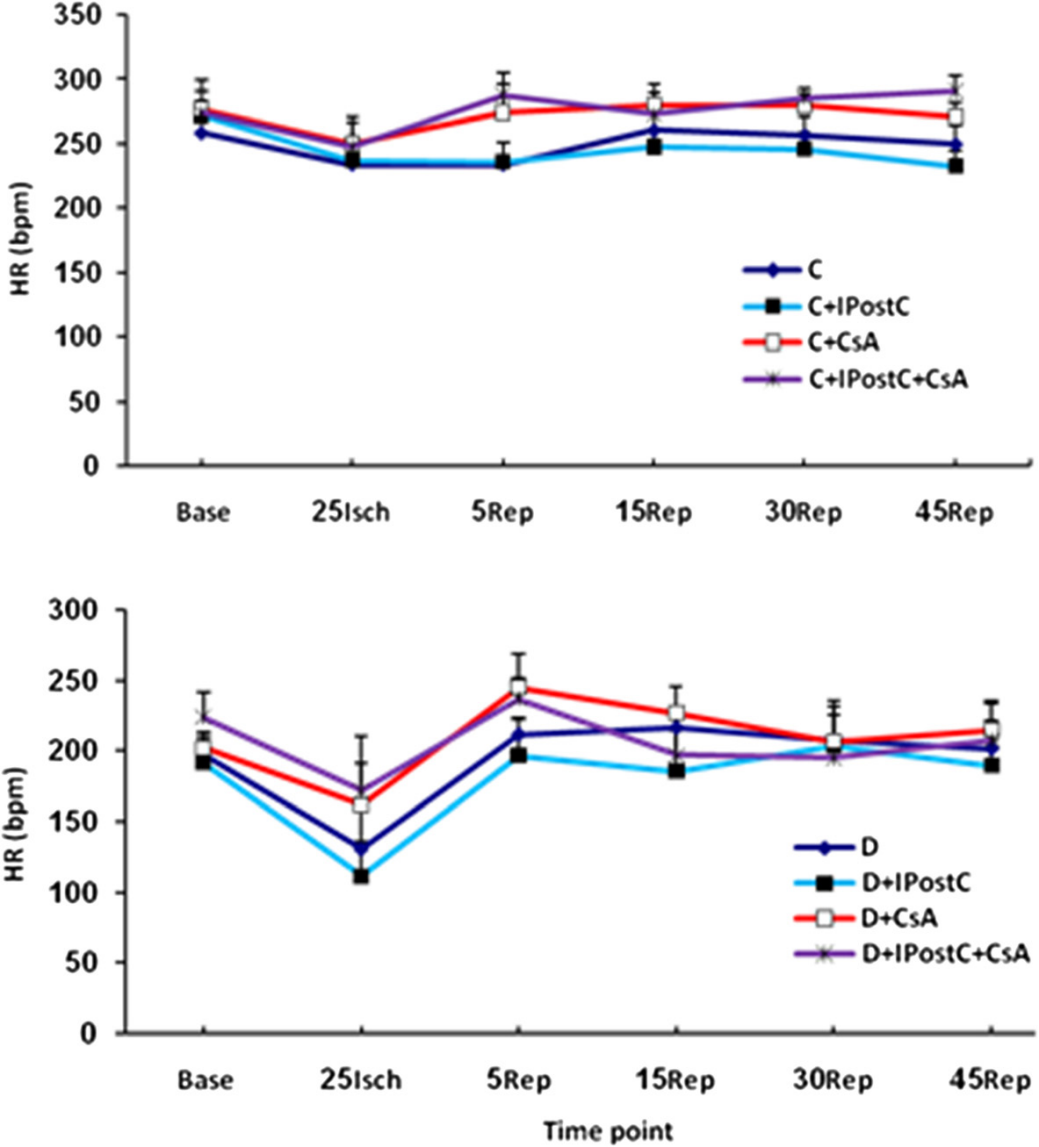
**The alterations of the heart rate (HR; in beats per minutes, bpm) in control (C; top) and diabetic (D; bottom) subgroups throughout the ischemia (Isch) and reperfusion (Rep) time.**

Diabetic hearts had lower HR than control animals during the experiment. In these hearts, the HR was reduced by 30 min ischemia and recovered through reperfusion. During I/R time, HR alterations in all groups were not statistically significant throughout the experiment. Additionally, there was no significant difference of this parameter among groups in ischemic and reperfusion phases of the control and diabetic groups (Figure 5).

### CF alterations during I/R

**Figure 6:**
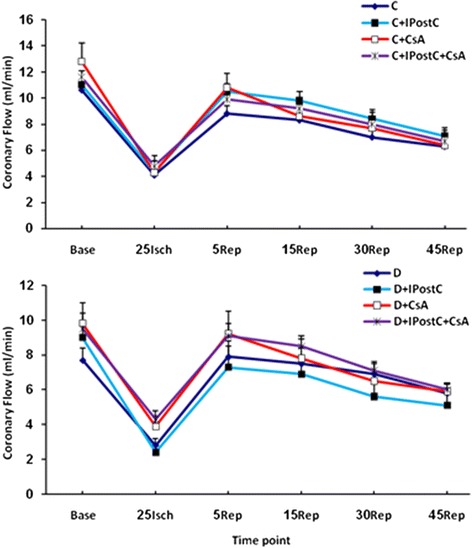
**The alterations of the coronary flow (CF; in ml/min) in control (C; top) and diabetic (D; bottom) subgroups throughout the ischemia (Isch) and reperfusion (Rep) time.**

Regional ischemia induced by occluding LAD reduced the coronary flow both in control and diabetic hearts. Although IPostC and/or CsA tended to increase the CF in reperfusion phase, there was no statistically significant difference in the CF in either diabetic or non-diabetic control subgroups throughout the experiment (Figure 6).

## Discussion

This study indicated that IPostC as a potent cardioprotective stimulus did not exert its protective effect in diabetic hearts. However, if the mPTP was inhibited simultaneously, the protection by IPostC might be restored in these diseased hearts. Because diabetes is the most common comorbidity of acute myocardial infarction in clinics and the prognosis of acute myocardial infarction with diabetes is not very good, this investigation has focused the postconditioning's protective effects on normal and diabetic myocardium.

IPostC is a clinically relevant and feasible strategy which protects the ischemic heart via several ways. The main pathways that have been suggested to contribute to IPostC protection are including Reperfusion Injury Salvage Kinases (RISK) pathway which involves the kinases PI3K/Akt and ERK1/2, and Survivor Activating Factor Enhancement (SAFE) pathway which acts through the activation of tumor necrosis factor-α, and JAK/STAT [4,16,19]. All these pathways which some of them is impaired in diabetic states [20], converge on the mitochondria via the modulation of several kinases such as glycogen synthase kinase-3β, Bax/Bad and the ε isoform of protein kinase C [16,19,21]. It is believed that the end effectors of IPostC in cardioprotection are inhibition of mPTP and/or opening of mitochondrial ATP-sensitive K^+^ channels [4,21,22].

mPTP remains closed during myocardial ischemia but opens due to Ca^2+^ overload and excessive production of reactive oxygen species during the onset of reperfusion which in turn, leads to disturbance in mitochondrial integrity and its function [22]. Long-lasting mPTP opening is followed by profound alterations of cellular bioenergetics that are considered irreversible [19]. Opening of the mPTP results in collapse of the mitochondrial membrane potential, and uncoupling of the respiratory chain. In addition, pore opening leads to an influx of solutes and water that causes mitochondrial matrix swelling and loss of critical electrochemical gradients. In this condition, the mitochondrial F1/FO ATP synthase, which it interacts with several mPTP regulators, actively hydrolyzes rather than synthesizes ATP, leading to inevitable cell death [19,23,24]. This is rapidly followed by ATP and NAD^+^ depletion and the greater increased mitochondrial volume which can ultimately rupture the outer membrane leading to release of inner membrane space contents such as proapoptotic factors, including cytochrome-c and thus also inhibits electron flow through the electron transport chain. These events are thought to be detrimental for myocardial reperfusion injury [10,21,22,24]. Among other components of mPTP, the involvement of F1/FO ATP synthase especially its c subunit in mPTP formation has recently been demonstrated and this has attained great interest in the field of myocardial I/R injury, because it links the mitochondrial function to its bioenergetics [23-26]. Recent evidences suggest that an uncoupling channel within the c-subunit ring of the F1/FO ATP synthase is the mitochondrial permeability transition pore [25].

In this study, IPostC alone could elicit protection in normal hearts and co-administration of CsA, as a potent mPTP blocker, with IPostC could not increase further the effect of IPostC in these hearts without underlying risk factor. This finding indicates that IPostC and CsA have no significant additive effect on each other in control animals, and the cardioprotection is achieved by enough inhibition of mPTP. However, in 8-week diabetic animals, we were unable to show any protection by IPostC. That is, IPostC in these animals neither restored the cardiac hemodynamic functions (such as LVEDP and RPP) toward baseline value nor reduced the infarct size and CK levels as compared to corresponding controls. Furthermore, CsA was also unable to induce such protective effects in diabetic hearts. There is no enough report about interaction of chronic diabetes with postconditioning; and our study was in this context. Wagner et al. [27] showed that IPostC fails to reduce infarct size in transgenic rats with metabolic syndrome. In addition, Przyklenk et al. [28] have reported that the diabetes abolishes the cardioprotection by postconditioning. However, the duration of type 1 diabetes in their study was 2 weeks which correlates with the acute phase of the disease and does not agree with chronic models. It is well-documented now that early and acute (four-week or less) and chronic (eight-week) phases of the diabetes respond differently to I/R insults and likely to protective interventions [6]. According to our results, the effects of postconditioning are abolished in ischemic hearts accompanied with underlying chronic diabetes. Nevertheless, restoration of protection to the ischemic hearts of diabetic patients is clinically important.

We showed in the present study that the protection of diabetic hearts against I/R injury is afforded by concomitant application of IPostC and CsA, which each of them separately produced cardioprotection only in healthy but not in diabetic animals. According to these findings, it seems that the effectiveness of IPostC in the diabetic hearts is likely not strong as enough as the normal state to activate endogenous cardioprotective pathways in an effective way. After bringing CsA into play, however, the effects of IPostC and CsA are added on each other, and their final cardioprotective effects are enhanced. During diabetes, there is a set of sub-cellular, hemodynamic and metabolic abnormalities, each may attenuate or impede the cardioprotective route of IPostC [9,29]. For example, greater oxidative stress and hyperglycemia can block the stimulatory effect of IPostC on different protective protein kinases or block the inhibitory effect of CsA and IPostC on mPTP. Therefore, the lack of cardioprotection in diabetic myocardium may be attributable, partly, to these abnormalities and alterations. Therefore, further research is needed to clarify the exact mechanisms of diabetes-induced loss of cardioprotection.

In conclusion, IPostC alone or CsA alone could not protect the diabetic myocardium against I/R injury. However, by increasing the potency of protective stimulus through co-application of two stimuli (IPostC plus CsA) can overcome the loss of protection in diabetic state. The loss of cardioprotection in diabetic heart can be partly reduced by targeting and sufficient inhibiting the mPTP.
